# Impact of routine FDG-PET/CT on locoregional treatment decisions in breast cancer patients receiving preoperative systemic therapy

**DOI:** 10.1016/j.breast.2025.104475

**Published:** 2025-04-08

**Authors:** Jetske L.B. Gunster, A. Marjolein Schrijver, Frederieke H. van Duijnhoven, Marcel P.M. Stokkel, Corrie A.M. Marijnen, Astrid N. Scholten

**Affiliations:** a Department of Radiation Oncology, Netherlands Cancer Institute-Antoni van Leeuwenhoek, Amsterdam, the Netherlands; b Department of Radiation Oncology, Leiden University Medical Center, Leiden, the Netherlands; c Department of Surgical Oncology, Netherlands Cancer Institute-Antoni van Leeuwenhoek, Amsterdam, the Netherlands; d Department of Nuclear Medicine, Netherlands Cancer Institute-Antoni van Leeuwenhoek, Amsterdam, the Netherlands

**Keywords:** Breast cancer, FDG-PET/CT, Preoperative systemic therapy, Regional radiation therapy, Axillary surgery

## Abstract

**Purpose:**

This study evaluates the clinical impact of routine FDG-PET/CT on locoregional treatment in a large cohort of breast cancer patients scheduled for preoperative systemic therapy (PST).

**Methods:**

Patients scheduled for PST were identified from a retrospective database between 2011 and 2020 at our hospital. All patients underwent staging by FDG-PET/CT prior to PST. The rate of regional upstaging by FDG-PET/CT compared to initial locoregional staging was assessed, as well as its implications on surgical and radiotherapeutic management. Logistic regression analysis was used to evaluate the correlation between clinical characteristics and regional upstaging by FDG-PET/CT.

**Results:**

Among 1228 eligible patients, FDG-PET/CT detected additional regional lymph node involvement in 145 patients (12 %). This resulted in treatment modifications for 140 patients (11 %), including changes to the axillary surgical approach in 27 patients (2 %), and adjustments to the postoperative radiation therapy plans in 115 patients (9 %). The majority of these modifications occurred in patients initially staged as cN1(1–3) (92/140). Clinical T stage was significantly associated with regional upstaging by FDG-PET/CT.

**Conclusion:**

FDG-PET/CT staging before PST frequently identifies additional regional lymph node involvement, significantly altering locoregional treatment strategies in the majority.

## Highlights

• The extent of regional lymph node metastases strongly impact breast cancer prognosis.
• The optimal method and timing for axillary staging in PST patients remain debated.
• FDG-PET/CT upstaged clinical N stage in 12 % of PST patients compared to conventional staging.
• Most upstaging occurred in patients initially staged with 1–3 positive axillary lymph node(s).
• Nearly all cases of upstaging led to significant treatment modifications.

## 1 Introduction

Locoregional lymph node involvement is a key prognostic factor in breast cancer, with 5-year survival rates decreasing from 99 % in node-negative to 86 % in node-positive disease, worsening with more extensive axillary or extra-axillary involvement [[1], [2], [3], [4]]. In patients undergoing preoperative systemic therapy (PST), both clinical and pathologic lymph node status are independent predictors of locoregional recurrence (LRR) and overall survival [[5], [6], [7], [8], [9], [10]].

Axillary staging before PST provides the baseline for optimizing both surgical and radiotherapeutic management. FDG-PET/CT is currently recommended for the detection of distant metastases in patients with stage IIB or higher [11]. However, FDG-PET/CT is also an effective non-invasive modality for axillary staging, with a high positive predictive value ranging between 85 % and 98 % [[12], [13], [14]]. Additionally, it provides the ability to quantify FDG-avid lymph nodes, offering valuable insights for treatment decisions [15].

Postoperative regional radiation therapy (RT) is recommended for patients at high risk for LRR, such as those with ≥4 positive axillary lymph nodes (ALNs), internal mammary lymph node (IMN) or periclavicular lymph node involvement, or T4 tumors [[16], [17], [18], [19], [20]]. These patients benefit from RT regardless of their pathologic response to PST, making it important to adequately identify these patients pre-PST [9]. Additionally, a risk assessment based on the number of involved ALNs can help guide the surgical approach. At our institute, the MARI (marking axillary lymph nodes with radioactive iodine seeds) procedure is used to pathologically stage the axilla after PST in cN+ patients [21]. Combining baseline FDG-PET/CT with MARI has led to an 82 % reduction in axillary lymph node dissections (ALND) [22,23].

All patients scheduled for PST at our institution undergo FDG-PET/CT as part of their pre-PST work-up, providing the opportunity to assess its clinical impact in an extensive cohort. This study aims to evaluate the implications of FDG-PET/CT on locoregional treatment decisions in breast cancer patients scheduled for PST.

## 2 Materials and methods

### 2.1 Study design and patient selection

This retrospective study was conducted at the Netherlands Cancer Institute-Antoni van Leeuwenhoek. Breast cancer patients scheduled for PST between January 2011 and December 2020 were identified from electronic medical records. Eligibility for the study required the availability of an FDG-PET/CT report. Patients with recurrent breast cancer or who had received treatment prior to FDG-PET/CT were excluded. The indication for PST was determined based on national guidelines [24], considering factors such as age, clinical stage, tumor size and histological type. For the current analysis, patients with distant metastases detected with FDG-PET/CT were excluded.

This study was approved by the Institutional Review Board of the Netherlands Cancer Institute-Antoni van Leeuwenhoek.

### 2.2 Initial locoregional staging

Initial clinical stage was based on physical examination, mammography, axillary ultrasound (AUS) and breast magnetic resonance imaging (MRI) for all patients. Lymph node status was evaluated using AUS, with fine needle aspiration cytology (FNAC) for pathologic lymph nodes. Breast MRI was used to assess tumor size at diagnosis.

### 2.3 18F-FDG-PET/CT

Prior to the FDG-PET/CT scan, patients were required to fast for 6 h and to have a blood glucose level below 10 mmol/L. A weight-adjusted dose of 180–240 MBq 18F-FDG was administered intravenously. Approximately 1 h later, FDG-PET/CT scanning (Philips Gemini TF Big Bore, Cleveland, OH, USA) was performed from the base of the skull to the groin, including a low-dose CT scan without contrast for attenuation correction and anatomical localization. Additional PET/CT images of the breast only were obtained in prone position to assess uptake in the primary tumor and locoregional lymph nodes.

### 2.4 Image interpretation

According to standard protocol at our institute, FDG-PET/CT scans were interpreted by an experienced nuclear medicine physician who had access to other imaging studies and relevant clinical data. We analyzed the data based on these interpretations.

Focally increased FDG-uptake observed exclusively on FDG-PET/CT, which did not correspond to physiologic patterns or pre-existing lesions, was classified as an additional lesion.

### 2.5 Regional upstaging

If necessary and feasible, additional lymph node lesions were further evaluated using targeting imaging procedures and, preferably, histopathologic confirmation. All cases were thoroughly reviewed and discussed in multidisciplinary meetings, where histological, radiological and clinical context were carefully considered before establishing final staging.

### 2.6 Data collection

At our institute, a modified clinical N stage is applied, differing from the American Joint Committee on Cancer (AJCC) TNM Staging. Specifically, patients with 1–3 positive ALNs on imaging are classified as cN1, in the current study denoted as cN1(1–3). Similarly, patients with ≥4 positive ALNs are classified as cN2a, denoted as cN2(4+). The remaining N stages align with the AJCC Staging system [25]. Definitions used in this study are summarized in Supplementary Table S1.

### 2.7 Standard axillary radiation therapy plans

In accordance with national guidelines [24], for cN0 patients axillary RT is indicated in case of ypN1 disease. For ypN1mi without the presence of risk factors (grade 3 tumors, lymphovascular invasion, tumors >3 cm), axillary levels I-II are included in the RT fields, whereas ypN1mi with risk factors or ypN1 involves axillary level I-IV. For cN1(1–3) patients, axillary RT to levels I-IV is indicated in case of ypN+ disease. In cN2(4+), and cN3 patients, axillary RT to levels I-IV is indicated regardless of response to PST. For cN2b patients, RT includes axillary levels III-IV and the IMN chain if ypN0, while levels I-II are added if ypN+. In case of stage cN3b, RT to axillary levels I-IV and the IMN chain is provided regardless of treatment response. A summary can be found in Supplementary Table S2. Lastly, for patients with tumors with direct extension to the chest wall and/or skin (cT4), all patients receive axillary level I-IV RT regardless of the clinical nodal status.

### 2.8 Standard axillary surgical treatment

Patients with cN0 or cN2b breast cancer undergo surgical axillary staging with sentinel lymph node biopsy (SLNB) following PST. For the remaining cN+ patients, a MARI procedure is performed [26]. In short, in case of cN1(1–3) disease, only a marked ALN is removed, and RT is administered if residual tumor is present. For patients with ≥4 ALNs before PST, the marked lymph node is removed; an ALND is performed only if residual tumor is found in the marked lymph node at frozen section.

### 2.9 Statistical analysis

Descriptive and explorative analyses were performed with the use of statistical software R version 4.3.3. Overall upstaging frequencies and proportions were estimated and reported as percentages. Associations between clinical characteristics and regional upstaging were assessed with univariable logistic regression analysis, with reference levels requiring a minimum of five events. The threshold for statistical significance was set at p < 0.05.

## 3 Results

### 3.1 Patient characteristics

Between January 2011 and December 2020, FDG-PET/CT data were available for 1471 breast cancer patients undergoing PST. Among these, 134 patients were excluded for reasons including recurrent breast cancer, prior surgical treatment or incomplete data. An additional 109 patients were excluded due to the detection of distant metastases on FDG-PET/CT (Supplementary Figure 1). Baseline characteristics are summarized in Table 1.

**Table 1:** Clinical and pathological baseline characteristics of all patients.

	All patients (n = 1228)	
**Age, mean (SD), years**	50	12
**Unilateral tumor**		
Yes	1182	96 %
No	46	4 %
**Unifocal tumor**		
Yes	854	70 %
No	372	30 %
Not applicable	2	<1 %
**cT stage prior to FDG-PET/CT**		
T0/is	7	1 %
T1	245	20 %
T2	757	62 %
T3	193	16 %
T4	26	2 %
**cN stage prior to FDG-PET/CT**		
N0	638	52 %
N1	429	35 %
N2	84	7 %
N3	77	6 %
**Histology**		
IDC	1056	86 %
ILC	138	11 %
Other	32	3 %
Missing	2	<1 %
**Subtype**		
HR+/HER2-	575	47 %
HER2+	312	25 %
HR-/HER2-	341	28 %
**Grade**		
1	53	4 %
2	597	49 %
3	541	44 %
Missing	37	3 %
**FDG-avid**		
Yes	1178	96 %
No	49	4 %
Missing	1	<1 %

Abbreviations: IDC invasive ductal carcinoma, ILC invasive lobular carcinoma, HR hormone receptor.

### 3.2 Initial locoregional staging

Based on initial clinical staging, 638 (52 %) of 1228 patients were categorized as cN0. A total of 429 patients (35 %) were classified as cN1(1–3), 83 patients (7 %) as cN2(4+), 1 patient (<1 %) as cN2b, 44 (4 %) as cN3a, 18 (1 %) as cN3b and 15 patients (1 %) as cN3c.

### 3.3 Locoregional staging with FDG-PET/CT

Following FDG-PET/CT, 145 patients (12 %) were found to have additional regional lymph node metastases. Histopathologic confirmation was performed in 22/145 patients (15 %), while additional targeted imaging was conducted in 4 patients (3 %). In the remaining 119 patients (82 %), FDG-PET/CT findings were considered sufficiently suspicious to confirm diagnosis without additional imaging and/or histopathologic confirmation. No patients were downstaged as a consequence of FDG-PET/CT. Axillary staging in prone compared to supine position did not result in concordant up- or downstaging [27].

### 3.4 Clinical implications of FDG-PET/CT

#### 3.4.1 Initial cN0 patients

In our cohort, 638 patients were initially staged as cN0. FDG-PET/CT upstaged 37 patients (6 %), with 19 patients (3 %) restaged as cN1(1–3), 2 patients (<1 %) as cN2(4+), 10 patients (2 %) as cN2b, 2 patients (<1 %) as cN3a, and 4 patients (1 %) as cN3b. Consequently, 27 of the 37 (73 %) upstaged patients became eligible for a MARI procedure instead of SLNB. Additionally, 18 patients (49 %) required regional RT regardless of their response to PST, with the IMN chain included in 14 patients (38 %). Notably, among the 8 patients upstaged to cN2(4+) or cN3, a positive MARI node could necessitate an ALND rather than RT alone.

Table 2 provides a detailed overview of upstaging rates by FDG-PET/CT, categorized by cN stage from initial locoregional staging.

**Table 2:** Summary of patients upstaged by FDG-PET/CT, categorized by clinical N stage based on initial locoregional staging. Percentages of upstaged patients are displayed alongside the absolute numbers, with the final column indicating the total number of patients upstaged per initial cN stage.

Initial imaging	Clinical N stage after FDG-PET/CT							Total(n)	N+(n)
	N0	N1(1–3)	N2(4+)	N2b	N3a	N3b	N3c		
**N0**	601	19 (3 %)	2 (0 %)	10 (2 %)	2 (0 %)	4 (1 %)	0	638	37 (6 %)
**N1(1**–**3)**	0	337	58 (14 %)	0	8 (2 %)	26 (6 %)	0	429	92 (21 %)
**N2(4+)**	0	0	74	0	5 (6 %)	4 (5 %)	0	83	9 (11 %)
**N2b**	0	0	0	1	0	0	0	1	0
**N3a**	0	0	0	0	37	7 (16 %)	0	44	7 (16 %)
**N3b**	0	0	0	0	0	18	0	18	0
**N3c**	0	0	0	0	0	0	15	15	0

**Total**	603	354	134	11	52	59	15	1228	145

#### 3.4.2 Initial cN1(1–3) patients

Of the 429 patients initially classified as cN1(1–3), FDG-PET/CT upstaged 92 patients (21 %). Specifically, 58 patients (14 %) were restaged as cN2(4+), 8 patients (2 %) as cN3a, and 26 patients (6 %) as cN3b. Among these upstaged patients were 6 patients with cT4 tumors, for whom regional RT was already indicated before FDG-PET/CT. For the remaining 86/92 patients (93 %), FDG-PET/CT determined the need for regional RT irrespective of treatment response, with 26 (28 %) of these patients also requiring inclusion of the IMN chain. In all 92 upstaged patients, a positive MARI node could mandate ALND instead of regional RT alone.

#### 3.4.3 Initial cN2 patients

Among 84 patients initially classified as cN2, FDG-PET/CT upstaged 9 patients (11 %) with previously undetected involvement of the infraclavicular lymph nodes (cN3a) in 5 patients and of the IMN chain (cN3b) in 4 patients. While all these upstaged patients already required regional RT including the periclavicular region, the 4 patients with IMN involvement required irradiation of the IMN chain as well.

#### 3.4.4 Initial cN3 patients

Within the 77 patients initially classified as cN3, 7 (9 %) were reclassified from cN3a to cN3b, necessitating adjustments to the RT plans to include the IMN chain.

#### 3.4.5 All patients

Of the 1228 patients, 140 patients (11 %) required treatment modifications following FDG-PET/CT. RT plans were adjusted in 115 patients (9 %), including 51 patients (4 %) who required inclusion of the IMN chain. As for surgical consequences, a total of 27 patients (2 %) required a MARI procedure instead of a SLNB. A total of 100 patients (8 %) were upstaged from initial cN0 or cN1(1–3) stage to either cN2(4+) or cN3. In case of a positive MARI node, these patients are candidates for ALND rather than receiving only RT to the regional lymph nodes.

### 3.5 Characteristics of upstaged patients

#### 3.5.1 cT stage

**Fig. 1:**
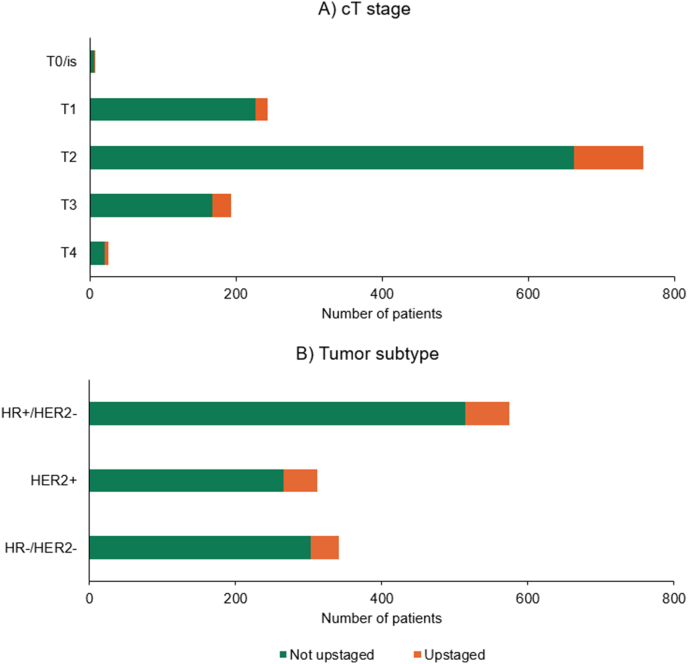
Distribution of upstaging by FDG-PET/CT in the overall cohort, stratified by A) initial cT stage and B) tumor subtype. The green bars represent the absolute number of patients with no change in cN stage following FDG-PET/CT, while the orange bars depict the absolute number of patients with regional upstaging.

In the total cohort, the majority of patients had cT2 tumors (n = 758), with 13 % upstaged by FDG-PET/CT (Fig. 1A). Upstaging rates were 7 % for cT1 and 13 % for cT3 patients. A small number of patients had cT0/is or cT4 tumors, with upstaging observed in 1/7 and 6/26 patients, respectively. In univariable analysis, a higher cT stage was significantly correlated with an increased risk of regional upstaging by FDG-PET/CT (Table 3).

**Table 3:** Univariable logistic regression analysis. Statistically significant values are displayed in bold formatting (p < 0.05).

	Univariable analysis		
	OR	95 % CI	p value
**Age**	1.00	0.99–1.02	0.710
**cT stage**			
0/is	2.22	0.25–19.48	0.471
1	ref	–	–
2	1.93	1.13–3.31	**0.016**
3	1.99	1.04–3.80	**0.037**
4	3.99	1.42–11.30	**0.009**
**Subtype**			
HR+/HER2-	ref	–	–
HER2+	1.46	0.97–2.20	0.072
HR-/HER2-	1.06	0.69–1.62	0.801
**Histology**			
IDC	ref	–	–
ILC	0.62	0.32–1.19	0.154
Other	1.34	0.51–3.54	0.555
**Grade**			
1	ref	–	–
2	1.32	0.51–3.42	0.572
3	1.24	0.48–3.24	0.657

Abbreviations: HR hormone receptor, IDC invasive ductal carcinoma, ILC invasive lobular carcinoma.

#### 3.5.2 Tumor subtype

Of the 575 HR+/HER2-patients in the cohort, 61 patients (11 %) were upstaged by FDG-PET/CT (Fig. 1B). Furthermore, upstaging occurred in 15 % of the 312 HER2+ patients and 11 % of the 341 HR-/HER2-patients. No significant association was demonstrated between tumor subtype and the risk of regional upstaging by FDG-PET/CT (Table 3).

#### 3.5.3 Other characteristics

The mean age of upstaged patients (51 ± 11 years) was similar to that of the rest of the cohort (50 ± 12 years). Upstaging rates for patients with grade 2 and grade 3 tumors were 12 % and 11 % respectively, compared to 9 % in grade 1 tumors. The cohort predominantly consisted of invasive ductal carcinoma (IDC) (n = 1056), followed by invasive lobular carcinoma (ILC) (n = 138) and other types (n = 32), with upstaging rates of 12 % for IDC, 8 % for ILC and 16 % for other types. No significant associations between the risk of regional upstaging and age, histological type and grade were found in univariable analysis (Table 3).

## 4 Discussion

This cohort study of 1228 breast cancer patients undergoing PST demonstrated that baseline FDG-PET/CT imaging led to the detection of additional regional lymph node metastases in 12 %, with treatment modifications in 11 %. Particularly in patients initially staged as cN1(1–3) by conventional locoregional imaging, FDG-PET/CT frequently identified more extensive lymph node involvement with considerable treatment modifications.

The application of systemic therapy in the preoperative rather than the postoperative setting is becoming standard practice for breast cancer patients requiring systemic treatment, enabling a reduction in the extent of both breast and ALN surgery [[28], [29], [30], [31], [32], [33]]. Aside from the importance of pathologic tumor response in guiding treatment, accurate radiologic staging of nodal involvement prior to PST is critical and serves as the baseline for regional lymph node management. Before the introduction of PST, regional RT strategies were primarily based on pathologic staging obtained from upfront surgery [19]. In the setting of PST, treatment effects obscure pathologic staging, complicating regional RT decision-making. Without regional RT, high-risk patients – those with ≥4 positive ALNs, IMN or periclavicular involvement, and tumors invading the chest wall and/or skin – remain at high-risk for LRR even after achieving a pathologic complete response [9]. Accurate pre-PST regional lymph node staging, particularly distinguishing N1(1–3) from N2(4+) or N3 disease, is therefore important for guiding RT decisions. Moreover, reliably identifying cN1(1–3) patients can reduce the need for ALND in these patients.

Axillary ultrasound (AUS), combined with fine needle aspiration cytology (FNAC) or core needle biopsy in case of suspicious nodes, is commonly used in the preoperative evaluation of breast cancer [34,35]. AUS is associated with low morbidity, high cost-effectiveness, and good accuracy in detecting ALN involvement [36,37]. However, it has its limitations. With a negative AUS, the likelihood of detecting advanced nodal disease (≥4 ALNs) is low, with a negative predictive value (NPV) of 95.5 % [38]. However, when the AUS is positive, its ability to exclude more advanced nodal involvement is limited, with a reported NPV of 58.5 % [38]. Breast MRI is primarily used to assess tumor size and extent for local treatment planning. As it typically includes a view of the axillary region, breast MRI may also offer the opportunity to evaluate regional lymph node involvement. However, previous studies have shown that preoperative AUS and standard breast MRI have comparable low diagnostic performance in differentiating between limited and advanced nodal disease, and both were deemed insufficient to accurately distinguish between these stages [39]. FDG-PET/CT outperforms AUS and MRI for locoregional staging [12], but its role in excluding advanced nodal disease in clinically node-positive patients has not been described in previous studies. However, with FDG-avid nodes strongly indicative for axillary metastases (PPV 96–98 %) and the ability to accurately quantify these nodes on FDG-PET/CT reconstruction images, it offers significant advantages in assessing the extent of regional lymph node involvement compared to AUS or breast MRI [12,40]. Previous research showed that FDG-PET/CT resulted in regional upstaging in 43/191 (23 %) PST patients compared to conventional methods, with therapeutic implications for all patients [15]. Similarly, Ng et al. reported that FDG-PET/CT led to upstaging and changes in regional RT plans in 15/139 (11 %) PST patients [41]. Both studies were conducted when PST was primarily used for locally advanced breast cancer in clinical trials. In our study, which includes a significant larger cohort of stage I to III PST patients, FDG-PET/CT consistently identified more extensive regional lymph node involvement, with notable clinical and prognostic implications in nearly all of them.

The majority of treatment modifications in our cohort occurred in patients initially staged as cN1(1–3). This aligns with previous findings that, while AUS and MRI can detect ALN involvement, they often fail to accurately determine its extent. Upstaging from limited nodal involvement (1–3 ALNs) to advanced nodal involvement (≥4 ALNs, periclavicular involvement) carries significant prognostic and therapeutic implications. It not only indicates the need for regional RT regardless of PST response, but may also necessitate ALND in case of a positive MARI node. Therefore, these patients are at risk of undertreatment without FDG-PET/CT. While current clinical guidelines recommend FDG-PET/CT for patients with AJCC stage 2b or higher – primarily for detecting distant metastases – our findings suggest that FDG-PET/CT also provides substantial benefits for stage 2a patients by influencing locoregional treatment decisions [11].

IMN metastases are particularly common in medial breast tumors and are associated with a poor prognosis, independent of the ALN status. FDG-avid nodes in the IMN chain are highly suggestive for metastases, with a PPV of 87.1 % [42]. In our cohort, 51 patients (4 %) had IMN metastases undetected by conventional imaging. Previous studies, involving smaller and more advanced breast cancer cohorts, reported unsuspected detection rates of 8–10 % [12,41,43].

To our knowledge, this retrospective study represents the largest cohort to date assessing the locoregional treatment implications of FDG-PET/CT in the PST work-up. However, this study has some limitations. The retrospective design and single-institution setting may introduce both selection and information bias, potentially affecting the generalizability of the results. Moreover, our analysis relied on standard clinical reads of FDG-PET/CT scans, interpreted by the treating nuclear medicine physician, introducing potential inter-reader variability. Even though all patients were thoroughly reviewed and discussed in multidisciplinary meetings before establishing a final diagnosis, histopathologic confirmation of lymph node metastases was limited in our cohort, and the possibility of false-positives should be considered. Furthermore, follow-up data were not included, preventing us from assessing whether these clinical implications translated into improved survival outcomes. To address this, future research should explore the long-term oncological impact of FDG-PET/CT in PST patients. Additionally, given the high costs of FDG-PET/CT and rising healthcare expenditures, the cost-effectiveness should also be a key consideration. Although a previous study indicated comparable costs between FDG-PET/CT and conventional imaging [44], the potential for incidental findings – often associated with full-body scans – may lead to additional follow-up investigations and increased costs [45]. Therefore, a comprehensive evaluation of the economic implications of FDG-PET/CT in breast cancer patients is warranted.

In conclusion, FDG-PET/CT had a significant impact on locoregional treatment for a substantial number of patients undergoing PST. As treatment paradigms are shifting with the growing use of PST, our study suggests that broadening the use of FDG-PET/CT to include stage 2a patients should be considered, given the high rate of regional upstaging and the resulting clinical implications in these patients.

## CRediT authorship contribution statement

**Jetske L.B. Gunster:** Writing – review & editing, Writing – original draft, Visualization, Methodology, Formal analysis, Data curation, Conceptualization. **A. Marjolein Schrijver:** Writing – review & editing, Data curation, Conceptualization. **Frederieke H. van Duijnhoven:** Writing – review & editing, Supervision, Resources, Methodology, Conceptualization. **Marcel P.M. Stokkel:** Writing – review & editing, Conceptualization. **Corrie A.M. Marijnen:** Writing – review & editing, Supervision. **Astrid N. Scholten:** Writing – review & editing, Supervision, Resources, Methodology, Conceptualization.

## Funding

We did not receive any grants from funding agencies in the public, commercial, or not-for-profit sectors.

## Declaration of competing interest

All authors have no relevant financial or non-financial interests to declare.
