# Supplementary material for: Impact of routine FDG-PET/CT on locoregional treatment decisions in breast cancer patients receiving preoperative systemic therapy
Source: Breast. 2025 Apr 8;81:104475. doi: 10.1016/j.breast.2025.104475 (PMC12138562; doi:10.1016/j.breast.2025.104475)
Supplement: Multimedia component 1 [file mmc1.pdf]

Supplementary

	Definition
**cN1(1-3)**	Presence of 1 to 3 positive axillary lymph nodes
**cN2(4+)**	Presence of ≥ 4 positive axillary lymph nodes
**cN2b**	Positive IMNs without involvement of axillary lymph nodes
**cN3a**	Presence of positive axillary and infraclavicular lymph nodes
**cN3b**	Presence of positive internal mammary and axillary lymph nodes
**cN3c**	Presence of positive axillary and supraclavicular lymph nodes

Supplementary Table S1. Definitions of clinical N stage as applied in routine clinical practice at our institute. IMNs internal mammary lymph nodes.

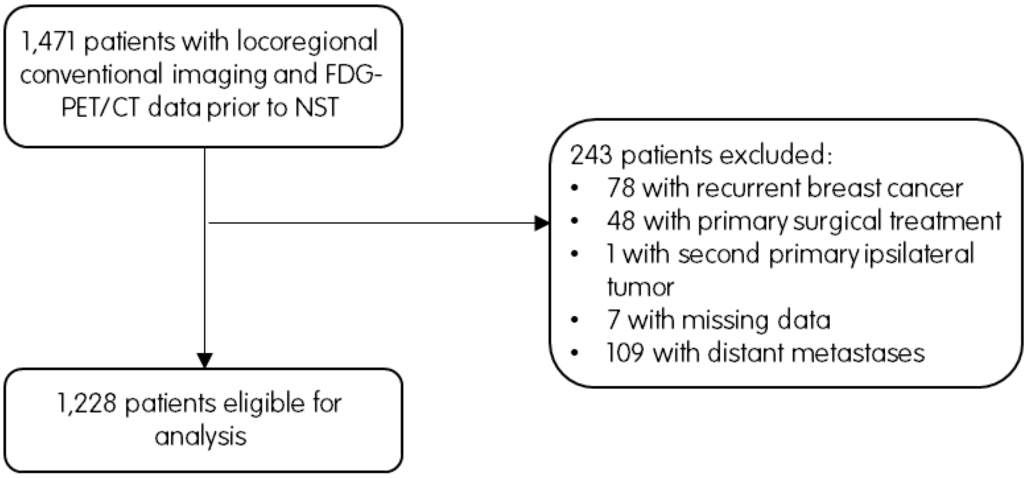

Supplementary Figure S1. Flowchart depicting the exclusion of patients from the current analysis.

N stage pre-PST	N stage post-PST	RT indication
**cN0**	ypN0	-
	ypN1mi (no RF)	Axilla level I + II
	ypN1mi (+ RF)	Axilla level I-IV
	ypN1	Axilla level I-IV
**cN1(1-3)**	ypN0	-
	ypN+	Axilla level I-IV
**cN2(4+)**	ypN0/+	Axilla level I-IV
**cN2b**	ypN0	Axilla level III-IV + IMNs
	ypN+	Axilla level I-IV + IMNs
**cN3a**	ypN0/+	Axilla level I-IV
**cN3b**	ypN0/+	Axilla level I-IV + IMNs
**cN3c**	ypN0/+	Axilla level I-IV

Supplementary Table S2. Summary of standard axillary RT plans used at our institute, based on clinical N stage and pathological N stage pre- and post-PST. Risk factors include grade 3 tumors, lymphovascular invasion, or tumors > 3 cm.

Abbreviations: PST preoperative systemic therapy, RT radiation therapy, RF risk factors, IMNs internal mammary lymph nodes.
